# Properties of realgar bioleaching using an extremely acidophilic bacterium and its antitumor mechanism as an anticancer agent

**DOI:** 10.1186/s40659-017-0122-y

**Published:** 2017-05-22

**Authors:** Peng Chen, Ruixiang Xu, Lei Yan, Zhengrong Wu, Yan Wei, Wenbin Zhao, Xin Wang, Qinjian Xie, Hongyu Li

**Affiliations:** 1 School of Pharmacy, Lanzhou University, Donggang West Road No. 199, Lanzhou, 730020 People’s Republic of China; 2 Gansu Key Laboratory of Biomonitoring and Bioremediation for Environmental Pollution, Institute of Microbiology, School of Life Sciences, Lanzhou University, Tianshui Road No. 222, Lanzhou, 730000 People’s Republic of China; 3 College of Life Science and Technology, Heilongjiang Bayi Agricultural University, Daqing, 163319 People’s Republic of China

**Keywords:** *A. ferrooxidans*, Anticancer effect, Bioleaching, RBS, Realgar

## Abstract

Realgar is a naturally occurring arsenic sulfide (or *Xionghuang*, in Chinese). It contains over 90% tetra-arsenic tetra-sulfide (As_4_S_4_). Currently, realgar has been confirmed the antitumor activities, both in vitro and in vivo, of realgar extracted using *Acidithiobacillus ferrooxidans* (*A. ferrooxidans*). Bioleaching, a new technology to greatly improve the use rate of arsenic extraction from realgar using bacteria, is a novel methodology that addressed a limitation of the traditional method for realgar preparation. The present systematic review reports on the research progress in realgar bioleaching and its antitumor mechanism as an anticancer agent. A total of 93 research articles that report on the biological activity of extracts from realgar using bacteria and its preparation were presented in this review. The realgar bioleaching solution (RBS) works by inducing apoptosis when it is used to treat tumor cells in vitro and in vivo. When it is used to treat animal model organisms in vivo, such as mice and *Caenorhabditis elegans*, tumor tissues grew more slowly, with mass necrosis. Meanwhile, the agent also showed obvious inhibition of tumor cell growth. Bioleaching technology greatly improves the utilization of realgar and is a novel methodology to improve the traditional method.

## Background

**Fig. 1:**
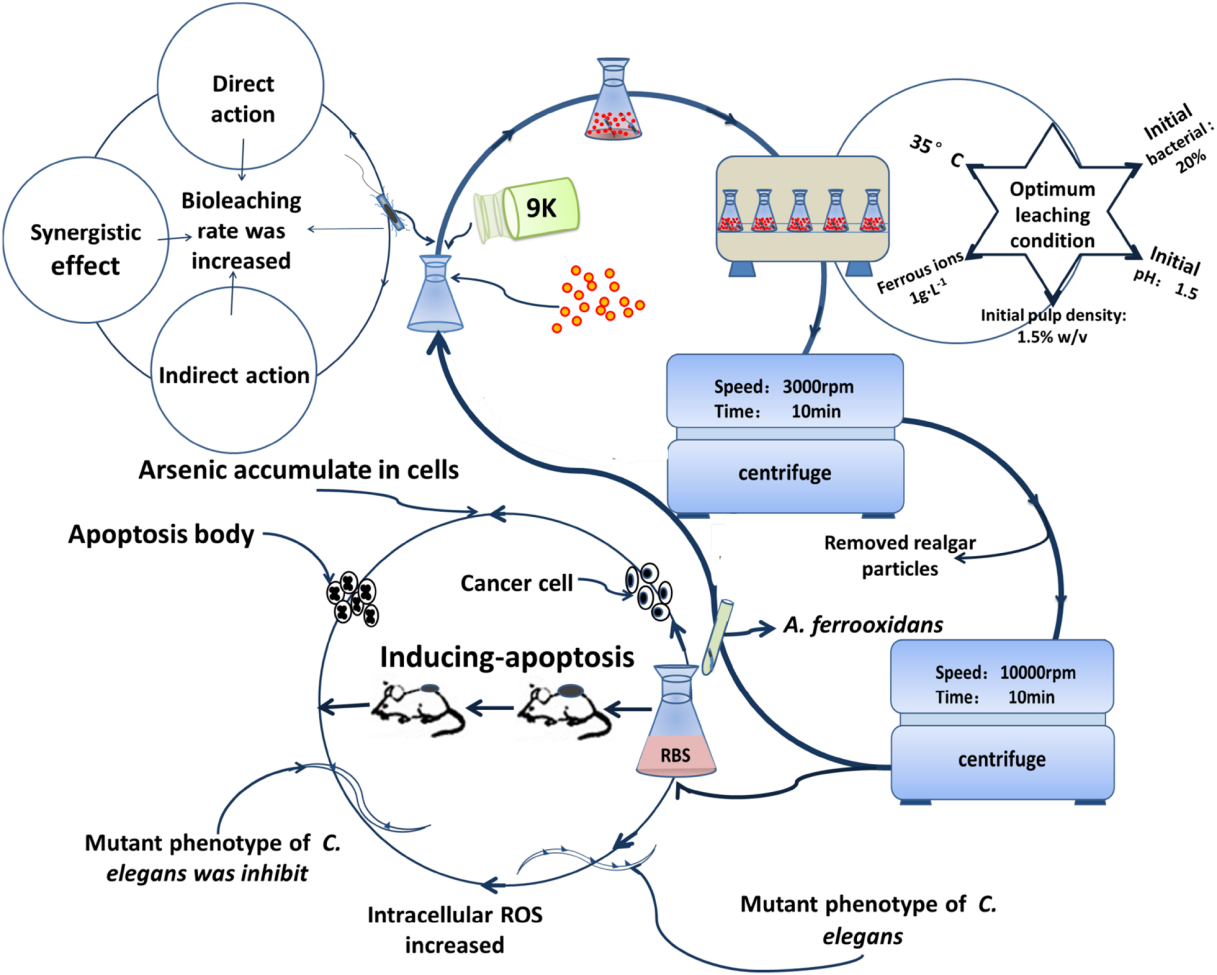
Bioleaching preparation and anticancer mechanism of realgar

Realgar, a traditional Chinese medicine, has been applied for more than 1800 years [1] to treat skin diseases by ancient Chinese doctors. Recent studies have indicated that realgar has an obvious anticancer effect in the clinic, especially on hematological cancer. Realgar research has been applied to the inhibition of the functions of acute promyelocytic leukemia (APL) and chronic early young granulocyte leukemia (CPL) [2]. However, realgar is one of a mineral agent with poor solubility. And it has been known as poisons since ancient times. Modern medical research demonstrated that it will cause a severe liver and kidney damage if intake realgar long-term [3]. Most importantly, it is well known that bioavailability of the agent is very poor. Previous study shows that just only 4% of total realgar in *Niu Huang Jie Du Pian* released into the blood system [4]. Those shortcomings above, such as high toxicity, poor bioavailability and low solubility, limit its application [5, 6]. To overcome these problems, a new technology called bioleaching is used to process realgar. This technology uses chemosynthetic bacteria, such as *Acidithiobacillus ferrooxidans* (*A. ferrooxidans*). The bacteria obtain energy by oxidizing ferrous ions and elemental sulfur. When *A. ferrooxidans* is used for bioleaching, the resolved rate of realgar is enhanced significantly and, as a result, the arsenic concentration in the solution increases markedly. Additionally, the pharmacological experiment confirms that the realgar bioleaching solution (RBS) has obvious antineoplastic activity in the K562 cell strain and H22 cell strain, similar to realgar. Meanwhile, the life expectancy of mice was extended and the mutant of *Caenorhabditis elegans* (*C. elegans*) was inhibited after treatment with RBS. This research suggested that RBS may be a promising agent against cancer with less toxicity and more effectiveness than raw realgar (Fig. 1).

## Clinical application of arsenic compounds

Many toxic natural product substances can be used as drugs to treat certain diseases. Some even have antitumor effects. More than 60 species have been shown to have efficacy [7]. Arsenic compounds, such as white arsenic (As_2_O_3_), red arsenic (As_4_S_4_) and yellow arsenic (As_2_S_3_), have been applied as a therapeutic agents for more than 2400 years [8]. The toxicity of these arsenic compounds led to them being utilized sparingly, with very few fixed doses in the ancient world. However, since the 1700s, arsenic has been widely employed in the clinic. The most prominent evidence is that white arsenic, as a first-line medicine, has been applied as an anti-leukemia treatment from the eighteenth to early twentieth century [9]. Today, the mechanism by which white arsenic treats APL is more clear. It can bind to PML-RARα (an oncogene protein that can enhance the growth of APL cells) to induce PML (promyelocytic leukemia protein) oligomerization [10]. White arsenic can induce APL cell apoptosis; the compound can downregulate the expression of apoptosis-regulated genes and enhance the concentration of reactive oxygen species (ROS) in APL cells. Overall, white arsenic induces apoptosis by controlling genes and ROS [11]. Furthermore, the induced effect is observed not only in blood cancer cells but also in additional solid cancer cells in vitro [12–14]. White arsenic can inhibit angiogenesis of cancer tissue when used to treat solid cancer [2, 15]. Importantly, Arsenic compounds did not lead to drug resistance compared with chemo-therapy drugs and selectively inhibit cancer cells with only minor side effects to normal cells [16, 17].

Although there is a long history of using white arsenic to treat APL, it was replaced by other anticancer medicines in the early 1970s due to its high toxicity [2]. As a similarly classified drug, realgar is less toxic and safer than white arsenic, but exhibits similar pharmacological activity. Thus, realgar has received scientific attention in recent decades. The chemical formula of realgar, a sulfide ore, is As_4_S_4_ [3, 18]. Because of its unique structure, realgar can bond to divalent lanthanide metallocenes and forms a double spherical structure in coordination chemistry [19]. Traditional Chinese medicine realgar powder is utilized with other medicinal materials to treat diseases in China (e.g., *Wan shen hua feng dan*, composed of realgar and other 13 components). The drug can considerably attenuate neurodegeneration by controlling the concentration of lipopolysaccharide (LPS, a key factor in nerve toxicity). The study indicates that microbial activation and the level of LPS in neurocytes are decreased. Nonetheless, the concentration of realgar in *Wan shen hua feng dan* should not be high (less than 0.3 mg/ml), as it does not show obvious toxicity to neurons [20]. Realgar also has an important role in *An Gong Niu Huang Wan*, *Niu Huang Jie Du Pian*,*Chijongdan* and other Chinese medicine [4, 21, 22]. Furthermore, compared with inorganic ions (As^3+^ and As^5+^), realgar showed less toxicity in mice, demonstrating that the agent is safer than inorganic ions when used to treat cancer [23]. However, realgar still has moderate toxicity. Neurotoxicity and a variety of extracellular amino acid changes are observed when tissues are treated with realgar [24, 25]. In addition, low solubility and poor bioavailability are two additional barriers in the application of realgar. Therefore, reducing toxicity and enhancing efficacy are two important process goals for realgar.

The common processing method for realgar is to grind it in solution [26]. This traditional approach can produce realgar powder at the micrometer scale. High energy ball milling, a new technique for processing realgar, can produce nano-meter scale powder [27]. Previous studies have shown that nanoscale realgar particles have higher bioavailability and toxicity than micron particles [28]. When realgar particles smaller than 150 nm the anticancer effect on cancer cells will increased significantly. The effect of realgar on cell survival and apoptosis in the ECV-304 cell line (human umbilical vein endothelial cell) have been proved it. In the research the cells were treat with four suspensions of realgar particles with diameters from 100 to 500 nm. Realgar particles were divided and marked RD100, RD150, RD200 and RD500 in the experiment. There are three obvious characters after treat with RD100 and RD150 for 8 h. Firstly, condensed chromatin, fragmented nuclei and blebbing of plasma membranes were markly visualized; secondly, the percentage of the apoptotic cells were 68.07, 49.62%, respectively; lastly, the level of arsenic distribution in cancer tissue was higher than other tissues or organs.

Data using realgar nano-particles to treat mouse melanoma skin cancer have shown that realgar at high concentrations (1–10 μM**/**l) can induce cancer cell apoptosis significantly, with less damage to normal cells [29]. Nanometer realgar was added in the medium to culture SiHa cells (Human uterine cervical cancer cell line) for 48 h. Proliferation of cells was inhibited compared to the control group and the characteristics of apoptosis were identified in cells. Further study demonstrated that realgar can down-regulate the transcription and translation expression of related genes; the levels of related protein in the experimental group were less than the control. Moreover, this phenomenon is observable to gradually increases with time and concentration [30]. When rat glioma cells were treated with nanometer realgar particles, the same effect was observed [31]. From the above, it is a fact of iprocessing raw realgar to nano-size can enhance the anticancer effect on tumor cells [32].

## Bioleaching of realgar by *A. ferrooxidans*

Recently, a novel technology called bioleaching was used to process realgar. This bio-technology was more effective and more responsive to the environment than the high energy ball mill. After bioleaching, the resolved rate of realgar was enhanced markedly and the arsenic concentration improved significantly. And the principle of biotechnology is similar to the hydrometallurgy in metallurgy.

Hydrometallurgy is commonly used in the metallurgical industry. Greater than 20% of copper was produced via hydrometallurgy in 2006 [33]. The technique uses an acidic or alkaline solution to leach metallic minerals [34]. After the process of leaching, target metallic ions were obtained from the equipment in the solution. In 1947, it was discovered that some acidophilic bacteria can live in acidic mine wastewater [35, 36]. Additionally, those bacteria can attach to mineral particles and greatly increase metal extraction through bio-oxidation [37]. The role of bacteria in leaching won the attention of scientists in the 1960s. Now 50 years later, bioleaching has become an essential technology in hydrometallurgy [38]. Mesophilic bacteria, such as *A. ferrooxidans*, *Acidithiobacillus thiooxidans* and *Leptospirillum ferrooxidans*, are frequently used to leach sulfide minerals [39]. *A. ferrooxidans* is the most commonly utilized microorganism in bioleaching [40], and its mechanism of leaching copper sulfide is known in detail [41].

Ferrous and elemental sulfur can be the energy source for *A. ferrooxidan* growth [42]. More importantly, wild-type bacteria can bear arsenic, and the InC of dimethyl arsenate for bacteria was 32 mM/l in 9 K medium, ([43–45]. It has been noted that there are obvious differences in tolerance to inorganic arsenic between wild and adapted bacteria [46, 47]. To obtain an adapted bacterial strain with high tolerance to arsenic ions, the bacteria were cultured in 9 K medium containing realgar [48, 49]. When the multiplication of bacteria reach to the plateau phase transfer them to fresh media containing a higher concentration of realgar [46]. After approximately 2 months domesticated, the arsenic resistance of the bacterium increased substantially [50].

Realgar (As_4_S_4_) is a sulfide ore in nature. Therefore, it have the same principle between using *A. ferrooxidans* to bioleach realgar and copper sulphide. Bacteria can significantly enhance the solubility of realgar and increase the arsenic concentration [49]. A series of single-factor experiments demonstrated the most suitable growth conditions for *A. ferrooxidans*.

Temperature is considered to be one of the most important factors for bacterial growth [51]. The best temperature for *A. ferrooxidans* growth is approximately 35 °C owing to the mesophilic nature of the bacteria [52]. Previous studies have shown that the concentration of arsenic ions significantly increased from 16.4 to 37.8 mg/l as the temperature changed from 15 to 40 °C [53, 54]. However, large amounts of jarosite are present in the medium if the temperature is above 35 °C. Jarosite can attach to the surface of realgar to and become a barrier to prevent the bio-oxidation effect of bacteria [55]. Therefore, formation of jarosite must be strictly controlled during bioleaching. Although rising temperatures can accelerate the dissolution of jarosite, they inhibit the activity of bacteria if the temperature is very high [56]. Meanwhile, *A. ferrooxidans* is a strict acidophile that grows in the pH range 1.4–3.0 [53, 54]. The initial pH values will affect microbial activity, which will significantly alter the bio-oxidation efficiency of ferrous to ferric iron [57]. The arsenic concentration in medium at pH 1.5 is 30 mg/l, similar to the group at pH 3. At pH values up to 3, ferrous ions will precipitate with other metal ions in the medium (jarosite), which will inhibit growth of bacteria [55]. As mentioned before, this precipitation greatly reduces the concentration of arsenic ions through physical adsorption [58]. Therefore, it is more efficient to bioleach realgar in a medium at pH 1.5 than at other pH values.

In the process of bioleaching, bacteria increase the solubility of realgar by oxidizing arsenic ions [59]. Therefore, the initial population of bacteria is another critical factor for bioleaching. Therefore, the 20% initial bacterial population is the better choice for bioleaching experiments. Four experimental groups were used to verify the effect of inoculation, ranging from 5 to 20%. A laboratory study on initial bacterial populations discovered that arsenic concentrations increased obviously after enhancing the bacterial population. The final arsenic concentration reached 115 mg/l when the initial bacterial population was 20%, far higher than that of the other three groups after 100 h [53, 54].

*Acidithiobacillus ferrooxidans* is a chemoautotroph that can utilize both ferrous and ferric ion as a source of energy for growth and reproduction. The ferrous ion concentration in the medium also influences the efficiency of bioleaching [60]. Therefore, it is necessary to add ferrous ions to the reaction system to maintain the growth of bacteria [48]. It can also be determined that the system that contains 1 g/l ferrous iron has the highest arsenic concentration compared to other groups. Laboratory studies have shown that too much ferrous iron in the leaching system does not enhance the rate of leaching significantly. Instead, it took on a negative role in the system [61]. Excessive amounts of iron compounds can adsorb arsenic ions in solution and are primarily responsible for the reduction of the arsenic concentration [61]. Moreover, high concentrations of ferric ions can inhibit the oxidation of ferrous ions by influencing the iron-oxidizing enzyme system of *A. ferrooxidans* [62], which will reduce the bioleaching rate. Thus, the best concentration of ferrous ion is 1 g/l in the bioleaching system.

The initial pulp density of minerals in bioleaching will affect the final metal concentration [63, 64]. Therefore, the pulp density of realgar is another important factor for bioleaching. At a 1.5% w/v initial pulp density, the bioleaching efficiency is highest because realgar is a traditional Chinese medicine that has shortcomings, including high toxicity and poor solubility. A high concentration of realgar (above 2% w/v) can inhibit the physiological activity of bacteria, leading to lower arsenic concentrations in the experimental group than in the control group [65]. At low realgar concentrations (below 0.5% w/v), the arsenic concentration under bioleaching is only slightly higher than that of the control group. Therefore, at a pulp density of 1.5% w/v, the concentration is 250 mg**/**l and the efficiency of bioleaching is more economical than at other pulp densities [53, 54]. Various factors affect the rate of bioleaching. Optimum conditions should be chosen to bioprocess realgar. In short, *A. ferrooxidans* was cultured in medium at pH 1.5 and 35 °C, with a 20% v/v inoculum, ferrous concentration of 1.0 g/l and 1.5% w/v pulp density. The arsenic concentration indicates that the bioleaching rate is efficient under these conditions.

## The mechanism of bioleaching of realgar

The bio-oxidation of *A. ferrooxidan* is the main factor that leads to increased realgar solubility. Bioleaching reactions consist of two stages that can be detailed in Eqs. (1) and (2) [49, 66]. The mechanism of bioleaching included direct, synergistic, and indirect action of *A. ferrooxidans*. First, the arsenic concentration was increased by the direct bio-oxidation of the attached bacteria, despite the fact that chemical groups, such as –OH and –NH, on the surface of bacteria can adsorb arsenic ions in solution. Second, the ferrous concentration and sulfur content in the solution will affect the bioleaching rate by influencing the activity of *A. ferrooxidans*, that is, the synergistic action between the bacterial and inorganic elements in the medium. Lastly, the action of *A. ferrooxidans* can catalyze the formation of inorganic compounds, such as Fe-containing compounds and S-containing compounds, which results in a decreased arsenic concentration, that is, indirect action of the bacteria. Fe-containing compounds can adsorb arsenic ions in solution; S-containing compounds form a layer on the surface of realgar particles that can inhibit the bio-oxidation of *A. ferrooxidans*.

1 $${\text{As}}_{ 2} {\text{S}}_{ 2} + {\text{14H}}_{ 2} {\text{O}}\mathop{\longrightarrow}\limits^{A. \, ferrooxidans} 2 {\text{H}}_{ 3} {\text{AsO}}_{ 3} + {\text{2HSO}}_{ 4}^{ - } + {\text{2OH}}^{ + } + {\text{18e}}^{- }$$

2 $${\text{H}}_{ 3} {\text{AsO}}_{ 3} + {\text{H}}_{ 2} {\text{O}}\mathop{\longrightarrow}\limits^{A. \, ferrooxidans}{\text{H}}_{ 3} {\text{AsO}}_{ 4} + {\text{2H}}^{ + } + {\text{ 2e}}^{{ - }}$$

### The direct action of bioleaching of realgar

Bacteria can influence the solubility of realgar through three diverse bioleaching mechanisms [67]. Previous research has revealed that *A. ferrooxidans* can attach to the surface of realgar particles and modify the surface, that is, direct action of *A. ferrooxidan.* When *A. ferrooxidans* is used to leach realgar changes on the surface, the particles can be confirmed by SEM and EDS. The result of testing showed various cracks at the surface of realgar particles. Meanwhile, the arsenic concentration significantly improved in the medium and similar changes in particles were not detect in the control group [68]. The bacterial effect may be the main reason for this phenomenon. Hence, the direct action of bacteria (attached bacteria) was the significant factor to improve the rate of dissolution in the process. That action was more significant than indirect action in the medium [69].

However, the direct action of bacteria also plays an unhelpful role in bioleaching. Some functional groups on the cell membrane of bacteria can absorb free arsenic from the medium [70]. This is a crucial factor leading to the reduction of the arsenic concentration in solution [71]. TEM analysis revealed that abundant arsenic was crowded around the bacteria in 9 K medium. This demonstrates that the bacterium can adopt released metal ions. Furthermore, Fourier transform infrared (FT-IR) spectra have demonstrated several differences between the experimental group and the control. The peak of the chemical group on the bacterial surface shifted, especially the operative groups –OH and –NH on the cell membrane [53, 54]. These functional groups may be This is a crucial factor leading arsenic ions [70]. Therefore, the biosorption of *A. ferrooxidans* is a critical factor for arsenic concentration decrease.

### The indirect action and synergistic effect of bioleaching of realgar

As mentioned, the direct action of bacteria was meaningfully influenced by the arsenic concentration. Meanwhile, the inorganic ion concentration, such as the ferrous ion concentration and sulfur content in the solution, will affect the bioleaching rate by influencing the activity of *A. ferrooxidans*. The biological activity of a bacterium will affect the bioleaching rate. The effects of inorganic ions and *A. ferrooxidans* are known as a synergistic effect. The indirect action is equally significant to the rate of dissolution. Inorganic compounds, such as iron compounds and sulfide, play a crucial role in indirect action [48].

### The effect of the iron-containing compound in bioleaching of realgar

Use of *A. ferrooxidans* to leach realgar in 9 K medium can be resolved as indicated in Eqs. (1) and (2). The catalysis of bacteria greatly increased the solubility of realgar. Furthermore, there is a more thorough explanation for the mechanism. When ferrous ions and chalcogenide ions are present in the medium, there are effects on the solution. These ions are the energy source for the bacteria, but ferrous irons and sulfur in the bioleaching system exert a significant influence on the rate of bioleaching. First, the ferrous ions used as the energy source of *A. ferrooxidan* can be detailed in Eqs. (3) [72]. From the formula above we know that bacteria can oxidize ferrous ion to ferric ion. Ferric ions generated by *A. ferrooxidan* can participate in the process of resolving realgar. Hence, the solubility of realgar greatly increases, as described in Eqs. (4) [73]. The concentration of arsenic ions was observed by scanning electron microscopy (ICP-AES), and the result showed that the arsenic concentration increased considerably.

3 $${\text{Fe}}^{{ 2 { + }}} + {\text{O}}_{ 2} + {\text{4H}}^{ + } \mathop{\longrightarrow}\limits^{A. \, ferrooxidan} 4 {\text{Fe}}^{{ 3 { + }}} + {\text{2H}}_{ 2} {\text{O}}$$

4 $${\text{As}}_{ 2} {\text{S}}_{ 2} + {\text{6Fe}}^{{ 3 { + }}} \mathop{\longrightarrow}\limits{{}} 2 {\text{As}}^{{ 3 { + }}} + {\text{2S}}_{{_{\text{surface}} }}^{ 0} + {\text{6Fe}}^{{ 2 { + }}}$$

5 $$H_{3} AsO_{4} + Fe^{3 + } \mathop{\longrightarrow}\limits^{A. \, ferrooxidan} FeAsO_{4} + 3H^{ + }$$

Moderate ferric ions were useful for efficient bioleaching. As an oxidant, ferric ions can promote the dissolution of realgar. These reactions can be characterized by Eqs. (3), (4), (5) and (6) [49]. Another effect of ferric iron was shown in a previous report. The study notes that ferric iron can reduce the arsenic concentration during absorption. Extreme concentrations of ferric ions in the medium play a negative role in bioleaching because they will co-precipitate with other metal ions and become a solid residue in the solution. The results of SEM and XRD suggest that this solid residue is jarosite. Jarosite precipitation may have occurred as described in Eqs. (7) [74], removing arsenic from adsorption in the solution [58]. Thus, arsenic concentration will decrease in the medium.

6 $${\text{HAsO}}_{ 2} + {\text{2Fe}}^{{ 3 { + }}} + {\text{2H}}_{ 2} {\text{O}}\mathop{\longrightarrow}\limits{{}}{\text{H}}_{ 3} {\text{AsO}}_{ 4} + {\text{2Fe}}^{{ 2 { + }}} + {\text{2H}}^{ + }$$

7 $$3 {\text{Fe}} + 2{\text{SO}}_{ 4}^{{ 2 { - }}} + {\text{X}}^{ + } + {\text{6H}}_{ 2} {\text{O}}\mathop{\longrightarrow}\limits{{}}{\text{X [Fe}}_{ 3} ( {\text{SO}}_{ 4} )_{ 2} ( {\text{OH)}}_{ 6} ] + {\text{ 6H}}^{ + }$$

X^+^ includes metallic ions, such as As^3+^, K^+^, Na^+^, NH_4_^+^ or H_3_O^+^.

### The effect of sulfide in bioleaching of realgar

As another inorganic compound, sulfide in the medium influences the efficiency of bioleaching. Sulfur compounds were produced gradually in the bioleaching system through the bio-effect of *A. ferrooxidans*. The reactions can be characterized by Eq. (8). Sulfur can attach to the surface of realgar to produce a sulfide layer that can prevent bacterial oxidation. Additionally, the presence of sulfur also affects the arsenic concentration by influencing cell activity. Specifically, sulfur was oxidized to sulfuric acid through the action of *A. ferrooxidans*. This reaction can be outlined by Eq. (9) [75]. The pH of the solution decreased due to the presence of sulfuric acid, conversely affecting the physiological activity of bacteria. Therefore, sulfur acid in the leaching solution also inhibited the efficiency of bioleaching.

8 $$2 {\text{As}}_{ 4} {\text{S}}_{ 4} + {\text{3O}}_{ 2} + {\text{6H}}_{ 2} {\text{O}}\mathop{\longrightarrow}\limits^{A. \, ferrooxidans} 4 {\text{H}}_{ 3} {\text{AsO}}_{ 3} + {\text{4S}}_{{_{\text{surface}} }}^{ 0}$$

9 $$2 {\text{S}}_{\text{surface}}^{ 0} + {\text{3O}}_{ 2} + {\text{2H}}_{ 2} {\text{O}}\mathop{\longrightarrow}\limits^{A. \, ferrooxidans} 2 {\text{SO}}_{ 4}^{{ 2 { - }}} + {\text{4H}}^{ + }$$

## The antitumor effect and application of realgar bioleaching solution (RBS)

Similar to arsenic compounds, when treating tumor cells with RBS (realgar bioleaching solution, also called realgar biotransform solution), the agent showed an obvious antitumor effect in vivo and in vitro and a significantly higher efficiency and safety than the raw realgar powder and other arsenic compounds [76]. It has better pharmacological activity effects and less toxicity than the realgar powder currently used in Chinese patent medicine. Hence, using RBS to prepare novel Chinese patent medicine will be the most valuable application in the future.

### The antitumor effect of RBS

The arsenic concentration of RBS is approximately 15-fold (10.40–156.23 mg/l) higher than the sterile control [6]. The experiment showed that RBS had a marked antitumor effect in vivo and in vitro. Flow cytometry (FCM) has shown the apoptosis-inducing effect of RBS on the Sarcoma-180 cell line (S_180_ cells). RBS remarkably induced cell apoptosis through inhibition of cell cycle pathways. This effect is expected to increase in a time- and concentration-dependent manner. Similarly, RBS has the exact same effect in Kunming mice.

**Fig. 2:**
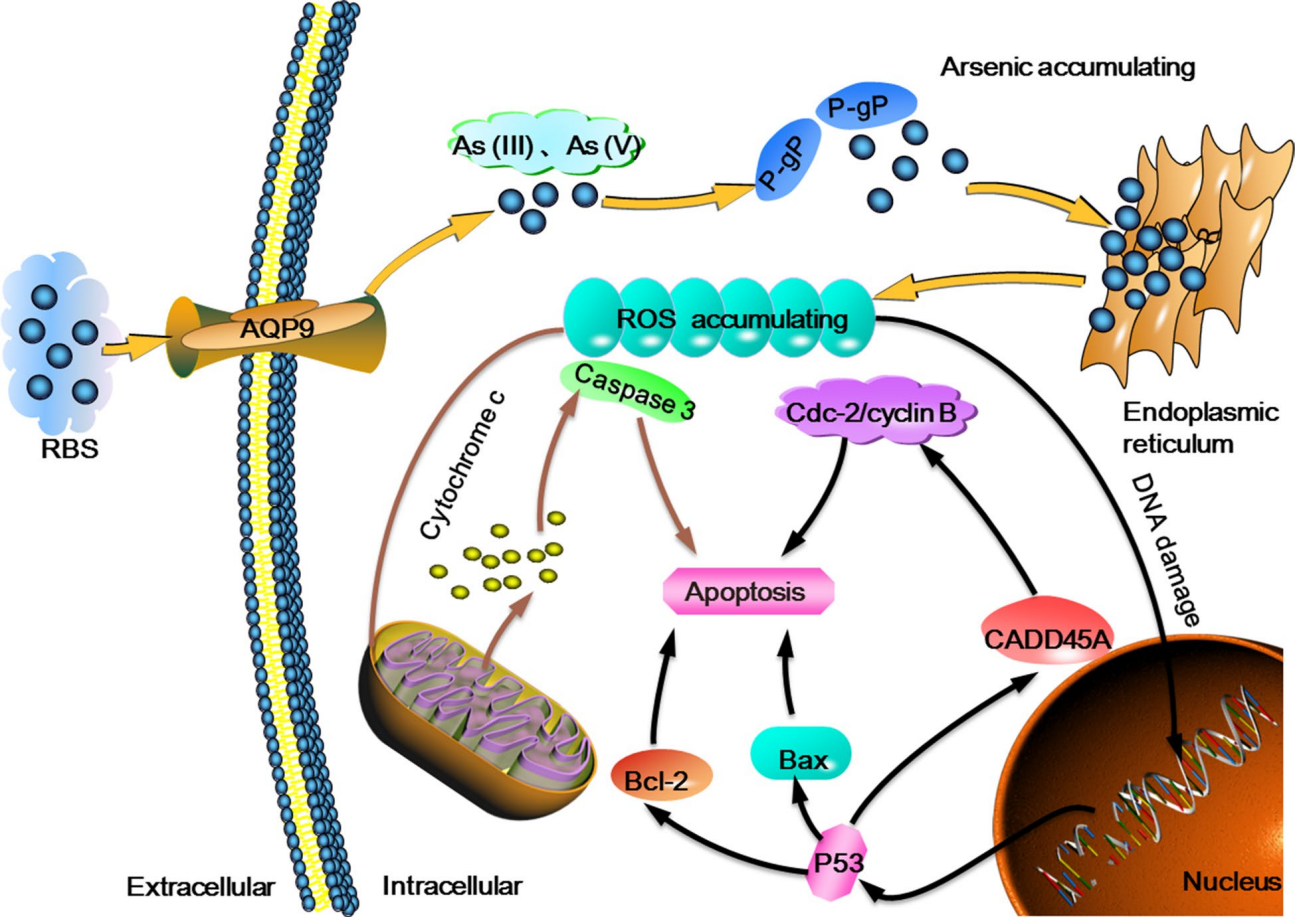
The mechanism of antitumor effect of RBS

RBS have a higher solubility and less toxicity when compare with raw realgar and ATO. Furthermore, the antitumor effect of it was enhanced, significantly (Table 1). The mechanism of antitumor effect of RBS is shown in Fig. 2, and then statistic of it was demonstrated in the Table 1. In the cancer cell line the parameters antitumor effect of RBS such as IC50 and apoptotic cells was higher than ATO. Moreover, in the animal model of mice the cancer inhibitory rate of RBS is greater than the kin drug. And in another animal model of *Caenorhabditis elegans* (*C. elegans*), the LC50 value of RBS was surpass the value of ATO which indicated that after bioleach the toxicity of RBS was less than other arsenic compounds such as ATO, raw realgar and the NPS. Meanwhile, as can be seen from the graph, there is a obvious increase in toxicity and bioavailability when processing raw realgar particles to the nano-size.

**Table 1:** The comparison of pharmacological activities among different arsenic agent in vivo and in vitro

Types	Models	Parameters	Realgar	NPS^f^	ATO	RBS	Reference
Cell lines	HepG_2_	IC_50_ in 72 h (μg/ml)	ND^a^	ND^a^	0.58 ± 0.08	0.43 ± 0.06	[10, 77]
	H_22_	Apoptotic rate^b^ (%)	ND^a^	ND^a^	12.8	15.8	[10, 77]
	H_22_	IC50 at 72 h (µM)	104.36	34.27	ND^a^	ND^a^	[27]
	H_22_	AUC_0→24_ (ng h/l)	1282.28 ± 586.23	4796.25 ± 479.885	ND^a^	ND^a^	[27]
	H_22_	t_½_ (h)	44.9 ± 18.1	53.3 ± 44.9	ND^a^	ND^a^	[27]
	H_22_	Biodistribution^c^ (ng/g)	320	610	ND^a^	ND^a^	[27]
	SiHa cells	Toxicity (g/kg)	3.2	ND^a^	0.033–0.039	ND^a^	[3, 30]
	SiHa cells	Bioavailability rate (%)	4%	ND^a^	High	ND^a^	[3, 30]
	C6	Apoptotic rate (%)	4.73% ± 0.46	26.02 ± 0.28	ND	ND^a^	[31]
	HeLa	IC_50_ at 72 h (µM)	8.69 ± 2.57	ND^a^	1.85 ± 0.54	ND^a^	[94]
	ECV-304	Toxic	More	Less	ND^a^	ND^a^	[32]
	ECV-304	Bioavailability	Low	High	ND^a^	ND^a^	[32]
	B16	Apoptotic rate (%)	ND^a^	11.4–54.95	ND^.a^	ND^.a^	[29]
	B16	Inhibitory rate^d^ (%)	ND^a^	ND^a^	22.70	27.0	[78]
	B16	Accumulating rate (%)	ND^.a^	ND^a^	36.6	71.50	[78]
	B16	t_½_ (h)	8.09 ± 2.27	10.13 ± 3.19	ND^a^	ND^a^	[1]
*C. elegans*	MT2124	LC_50_ (μg/mg)	ND	ND^a^	55	81	[76]
	S_180_-bearing	Inhibition rate (%)	39.5	53.14	ND^a^	ND^a^	[1]
	H22-bearing	Inhibitory rate (%)	ND^a^	ND^a^	41.87	42.78	[10, 77]
Mice	H22-bearing	Extending rates^e^ (%)	ND^a^	ND^a^	91.96	118.80	[10, 77]
	Healthy mice	LD_50_ (g/kg)	3.2	ND^a^	0.033–0.039	ND^a^	[4]
	Healthy mice	Bioavailability rate (%)	4%	ND^a^	ND^a^	ND^a^	[4]

*ND* no data

^a^The figures is not given in the paper

^b^The percentage of the apoptotic cells

^c^The arsenic accumulation in tumor

^d^The cancer cells growth inhibitory rate

^e^The mice life extending rate

^f^Realgar nanometer particles

In order to prove the antitumor effect of the RBS in vivo the cancer cells was transplanted to the Kunming mice. Then, treat the bearing mice with RBS in difference concentration respectively. Two obvious curative effects were discovered in mice: the weight of tumor growth was less than that of the normal group, and the tumor inhibition rate (at 3.24 mg/kg) reach to 32.2%; additionally, RBS can extended mouse lifetimes, and the life extending rate (3.0 mg/kg) is 118% [77]. More importantly, the arsenic accumulating rate in tumor is 71.5%, and it is greater than other organs or tissues such as in the lung (23.6%) and liver (59.53%). Therefore, the result indicating that RBS may be a selective high affinity tumor drug in clinical trials [78]. It was known that one of important indicators for cell apoptosis is that there will be a large number of cells remain in the G1 phase of the cell cycle. The result of FCM shown that cancer cells in the sub-G1 phase take up a higher percentage (24.3%) when treated with RBS at 1 μg/l. Thus, the apoptosis of cancer cells led by RBS might slow the proliferation of cancer cells and prolonging the life of the mice. [77]. Additional, RBS has the same effect for other cancer cells, such as H22 cancer cells in mice [1].

*Caenorhabditis elegans* is a model organism that is used to measure the pharmacological activity of anticancer drugs. RBS can suppress the ras gene in *C. elegans*. Ras gene, one of the characteristic gene relate with cancer. The Ras/MAPK signaling pathway has associated with proliferation, growth, and other living activity of the germ line [79, 80]. If ras gene activated excessively in *C. elegans* it will lead to the wild type become the multivulva (Muv) phenotypes [81]. Therefore, the types of worms fall into two categories: wild type and multivulva (Muv) phenotypes. These worms were cultured to adults after 45 days, the result shown that when *C. elegans* absorbed RBS into their body the mutant phenotype was affected and the brood size decreased significantly. The research point out that RBS can inhibit the excessive expression of genes and was be most probably in a promising anti-tumor drug candidate. Meanwhile, RBS has less toxicity in *C. elegans* compared to ATO. The LD_50_ value of RBS is 81 μg/mL compare with the values of 55 μg/ml of ATO [76].

The mechanism of apoptosis of RBS was verified when utilized in some tumor cells. RBS displayed significant activity against CML. RBS shown an obvious inhibiting effect in K562 cells (K562:CML cell line) in vitro cell culture experiment The research suggested that RBS might be a promising agent against CML of apoptosis and showed less toxicity and more effectiveness against K562/ADM cells compared with arsenic trioxide (ATO) and raw realgar [82]. The concentration of intracellular protein such as P-glucoprotein (P-gp) and AQP9 have obvious changes. That may be the main reason lead the RBS inhibit the growth of the cancer cells. P-gp can regulate the arsenic resistance of tumor cells, and the AQP9 expression can transport arsenic compounds and regulate the arsenic sensitivity of the cells [83, 84]. Additional studies found that RBS can down-regulate the over expression of MDR1 mRNA and P-gp and up-regulated the expression of AQP9 in the K562/ADM cell line. Those level changes of protein contributed to increased arsenic bioavailability in cancer cells, and led to arsenic accumulating in K562/ADM cells. The arsenic concentration accumulating will increased the level of intracellular ROS. Therefore, the mechanism of RBS can inhibit excessive activation of ras by enhancing reactive oxygen species (ROS) in *C. elegans* or cancer cells. There is a recognizable increase in the levels of ROS in cancer cells, and the level of intracellular ROS accumulation in the experimental group worms is two times (6.4 μg/ml) that of the control [85]. which shows that the higher level of ROS in cancer cell plays an important role in the process of apoptosis [86]. Further study showed that accumulated concentration of ROS will lead to cancer cell death if it exceeds a level that the cancer cells cannot bear [87].

A more detailed description is that an arsenic ion in RBS was transported into intracellular by AQP9 (one of the aquaporin water channel family), When the arsenic ions transported into cytoplasm the expression of AQP9 was enhanced. That change increased the uptake of arsenic ions. Another kind of change is that the level of P-gp (a protein relate to the excretion of arsenic species) was suppressed which attenuate the metabolism of arsenic in cancer cells particularly. Those above variation of the cells clearly contributed to the increase of arsenic concentration in the cancer cell. And most of the arsenic was transported to the endoplasmic reticulum.

The mechanism of RBS was inducing of apoptosis by increasing the concentration of ROS in the cancer cells. As stated previously, arsenic ions have been converted to the endoplasmic reticulum. The increased of arsenic could inhibit the cellular antioxidant defense system which led to abundant ROS accumulating. On one hand, the ROS accumulating can led the production of DNA damage. Once that happens, the gene of P53 and CADD45A will be activated which bring out the levels of protein expression increase in some certain apoptosis promoting proteins such as Bax, cyclin B1 and Cdc2, and the decrease in anti-apoptosis protein such as Bcl-2. On the other hand, ROS accumulating can make the effect of depolarization on the outer membrane of mitochondrial by the changes of Bax and Bcl-2. It will lead to the release of cytochrome c into the cytosol. For this reason, the level of pro-caspase-3 (Caspase-3): The important enzyme in the process of cell apoptosis) decreased and the cleaved-caspase-3 increased. Meanwhile, the obvious enhancement of the level caspase-3 was also observed in RBS treatment group. In short, the above two findings suggest that the apoptosis of the cancer cell by RBS via controlling the activation of P53 and other related proteins [88]. The possible cause of the gene are turned on may be the accumulation of ROS in the cancer cells. This mechanism above show that why the RBS has an obvious antitumor effect both in vivo and in vitro. And it can function as an agent in the treatment of cancer.

### The local application of realgar RBS

Compared with traditional Chinese patent medicine, the novel medicine containing RBS has great advantages in application. First, the pharmacological activity was greatly improved and side effects, such as toxicity and gastrointestinal tract irritation, were significantly reduced, [78]. Chinese patent medicines are advocated around the world. However, traditional Chinese medicines commonly contain toxic ingredients, such as heavy metal ions, which limit their application and export [89]. Realgar, a mineral medicine with low solubility and high toxicity, is a commonly used material to prepare traditional Chinese medicine. There are many Chinese patent medicines, such as *Niuhuang Jiedu Wan*, *Jufang Zhibao San*, *Fufang Qingdai Pian*, *Angong NiuHuang San*, *Wansheng Huafeng Dan*, among others, containing realgar [90–92]. Because they contain a large dose of realgar, these medicines showed high toxicity and obvious gastrointestinal tract irritation in clinic [93]. Replacement of realgar with RBS in prescriptions will reduce the application of realgar, leading to better control of the concentration of. RBS showed similar pharmacological activity to the original medicine containing raw realgar powder in vivo and in vitro [78]. Moreover, the anti-tumor mechanism of RBS was similar to other arsenic-based drugs such as white arsenic and yellow arsenic [94]. The novel medicine containing RBS would be safer and the discomfort associated with the drugs would be reduced [76, 82]. Therefore, RBS can substitute realgar powder and be used to prepare novel Chinese medicines in the future.

## Conclusion

The solubility of realgar was increased significantly when using *A. ferrooxidans* for bioleaching. *A. ferrooxidans* was cultured in medium at pH 1.5 and 35 °C, with 20% v/v inoculum, a ferrous concentration of 1.0 g/l and a 1.5% w/v pulp density. Under these conditions, the efficiency of bioleaching was highest and the function of bacteria was maximal. Furthermore, RBS showed an obvious antitumor effect in vivo and in vitro through induction of apoptosis. More importantly, RBS had a higher efficiency and safety than raw realgar powder and other arsenic compounds.

## Abbreviations

APL: acute promyelocytic leukemia
CPL: chronic early young granulocyte leukemia
RBS: realgar bioleaching solution
PML: promyelocytic leukemia protein
ROS: reactive oxygen species
LPS: lipopolysaccharide
